# Electrospinning of animal-free derived collagen-like protein: Development and characterization of VECOLLAN®- nanofibers for biomedical applications^☆^

**DOI:** 10.1016/j.ijpx.2025.100398

**Published:** 2025-09-19

**Authors:** Christoph Krauss, Maria Montero Mirabet, Jian-Feng Zhang, Karsten Mäder

**Affiliations:** a Institute of Pharmacy, Martin Luther University Halle-Wittenberg, Kurt-Mothes-Str. 3, 06120 Halle (Saale), Germany; b Evonik Operations GmbH, Research Development & Innovation, 64293 Darmstadt, Germany; c Evonik Corporation, Birmingham Laboratories, Birmingham, AL 35211, United States

**Keywords:** Collagen-like protein, Electrospinning, Non-animal-derived, Crosslinking, Drug delivery, Fibers, Wound healing

## Abstract

This study investigates the potential of VECOLLAN®, a recombinant, novel, non-animal-derived collagen-like protein, for use in electrospinning applications relevant to biomedical and drug delivery sectors. Given the limitations of animal-derived collagens, including immunogenicity and variability, VECOLLAN® offers a promising alternative due to its biotechnological production and non-immunogenic, non-allergenic, and non-inflammatory properties. We optimized the electrospinning parameters for VECOLLAN® and examined the effects of a novel coaxial crosslinking approach on the dissolution and disintegration behaviors of the resultant fibers. Our results demonstrate that VECOLLAN®-based fibers can achieve varying degrees of water insolubility, influenced by crosslinker concentration and type of crosslinker. Additionally, the fibers exhibit distinct swelling behaviors. With the addition of hyaluronic acid, the water absorption capacity could be increased. We investigated the distribution of silver nanoparticles within the fibers, confirming the homogeneity of the coaxial electrospinning process. Mechanical tests revealed that increased crosslinker concentrations lead to greater stability and rigidity, while elastin incorporation improved elongation properties. This study lays the groundwork for developing electrospun fibers made from a non-animal-derived collagen-like protein, highlighting the potential for applications in drug delivery and tissue engineering. Future research should focus on assessing the biocompatibility of these fibers further to explore their utility as drug carriers or cell scaffolds. Overall, our findings underscore the promising properties of VECOLLAN®-based fibers in advancing innovative solutions in the biomedical and drug delivery sectors.

## Highlights

• Developed non-animal-derived collagen-like protein-based fibers with tailored water absorption properties.
• A coaxial setup was employed to achieve in situ crosslinking during the process.
• Investigated the impact of crosslinking on fiber stability and elasticity.
• Highlighted potential applications in drug delivery and wound healing.

## Graphical Abstract

**Figure:**
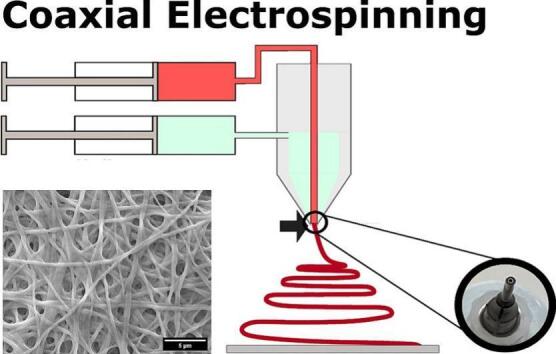

## 1 Introduction

Collagen is an important material for biomedical and drug delivery applications. It is highly biocompatible and biodegradable, has a high mechanical strength, and guarantees the structural integrity of tissues. Its versatile properties make collagen a valuable biomaterial used across multiple fields (Muthukumar et al., 2017; Rezvani Ghomi et al., 2021).

The market size for collagen used for medical devices and research was 111.5 million USD in 2023, with an estimated annual growth rate (CAGR) of 4.6 % until 2030. The CAGR (2023−2030) for pharmaceutical products is 6.0 %, reaching 1548.4 million USD in 2030 (Rizvi et al., 2023). Most of the collagen currently used in the medical device sector is animal- or allograft-derived collagen (Abedi et al., 2024).

Animal-derived proteins used in biomedical applications have several drawbacks. They can trigger immune responses, exhibit high biological variability, and raise ethical concerns about animal welfare (Duarte et al., 2023). Specifically, animal-derived collagen has the potential for contamination with viruses or other infectious agents. Moreover, the purification process for specific types of collagen is technically difficult because animal-derived collagen typically contains a mixture of different subtypes (Liu et al., 2022). Considering the drawbacks of animal-derived proteins, exploring and establishing non-animal-derived alternatives is prudent and necessary (Rezvani Ghomi et al., 2021). Non-animal-derived proteins are an attractive alternative to animal-derived proteins because they offer superior biological safety (Schellekens and Casadevall, 2004). Furthermore, recombinant protein synthesis technology allows the precise and reproducible creation of specific materials by utilizing cellular machinery, eliminating compositional variation, and achieving molecular-level consistency (DiMarco and Heilshorn, 2012).

Several recombinantly produced molecules are investigated for their potential use in the biomedical sector, for example, in tissue engineering or drug delivery applications. Examples are elastin, which plays a key role in the extracellular matrix (Corchero et al., 2014), and hyaluronic acid, found in various tissues and the skin (Izawa et al., 2011). Also, plant-based proteins have garnered significant interest due to their abundance, customizable properties, biodegradability, biocompatibility, and various biological activities. Among these, corn, soy, and wheat proteins are the most extensively studied (Adamu et al., 2021; Popov Pereira et al., 2021; Wen et al., 2022). For example, Marzalik and coworkers presented plant protein-based electrospun fibers that are promising biomaterials for skin regeneration and wound healing, exhibiting biocompatibility, antimicrobial effects, and anti-inflammatory activity, with a focus on zein, soy, wheat gluten, and pea protein in tissue engineering applications (Marszalik et al., 2025).

Due to collagen's intrinsic properties and significant market potential, a non-animal-derived variant is desirable.

Recombinant collagen-like proteins (CLPs) offer the opportunity for a rational design with tailored characteristics, potentially enhancing the effectiveness of therapeutic applications (DiMarco and Heilshorn, 2012). A novel, non-animal-derived, collagen-like protein is VECOLLAN® (Evonik Industries AG, Germany). VECOLLAN® has a molecular weight of 22.8 kDa and exhibits properties such as being non-immunogenic, non-allergenic, and non-inflammatory while promoting cell proliferation and wound healing (Born and Krauter, 2025; Weber, 2024). Due to its properties, VECOLLAN® is also a promising candidate for use in the sensitive field of parenteral drug delivery.

Electrospinning is a versatile technique used in various biomedical and drug-delivery applications (Keirouz et al., 2023) and a gentle way to process thermosensitive substances, such as proteins (Meli et al., 2010). Electrospun nanofibers have diverse favorable properties, such as a high surface-to-volume ratio and the ability to form scaffolds that mimic the extracellular matrix (Zulkifli et al., 2023). It provides scalability and simplicity and uniquely combines a high fabrication rate, broad material compatibility, and low cost (Rostami et al., 2023).

Therefore, the current study aims to (1) explore the potential and limitations of the non-animal-derived protein VECOLLAN® for electrospinning. Suitable parameters of the electrospinning formulation and processing should be identified. In addition, (2) the impact of crosslinking on the dissolution and disintegration behavior of the fibers should be investigated. Furthermore, (3) a material-scientific examination is conducted.

## 2 Materials and methods

### 2.1 Use of the word “fibers”

In some experiments, instead of individual fibers, a large number of stacked fibers forming a nonwoven mat were used. For the sake of simplicity, the term “fibers” will be used throughout the entire manuscript.

### 2.2 Materials

VECOLLAN® was provided by Evonik Industries AG, Germany. Polyethylene oxide (PEO) with a molecular weight of 400 kDa was purchased from Merck KGaA, Germany. Hyaluronic acid (610 kDa) was purchased from Matexcel, USA. Elastin (10 kDa) was purchased from Matrihealth GmbH, Germany. 4-(4,6-Dimethoxy-1,3,5-triazin-2-yl)-4-methylmorpholinium Chloride (DMTMM) was purchased from TCI Deutschland GmbH, Germany. 4-Arm-polyethylene glycol-succinimidyl-glutarate-ester with a molecular weight of 10 kDa (PEG 4-Arm CL) was purchased from JenKem Technology USA Inc. 5 nm Silver nanoparticle suspension 2200 ppm was purchased from NanographeneX, United Kingdom. Acetone EMSURE® was purchased from Merck KGaA, Germany.

### 2.3 Electrospinning

Electrospinning was conducted using a FLUIDNATEK LE-50 electrospinning equipment (BIOINICIA FLUIDNATEK SLU, Spain). The electrospun fibers were made from VECOLLAN® and PEO. The composition of the final formulation is 120 mg/mL VECOLLAN® and 18.6 mg/mL PEO. In one experiment, hyaluronic acid was added, and in another, elastin. The solvent mixture for all electrospinning formulations consisted of double-distilled water/acetone [9:1 (*v*/v)]. The fibers were produced with or without crosslinkers, depending on the experiment. The applied voltage to generate the fibers was 14–22 kV. The negative voltage on the flat collector was constantly −5 kV. The temperature in the climate chamber of the Fluidnatek LE-50 was set to 25 °C, and the relative humidity was set to 40 %for all experiments. The spinneret to collector distance was 22.5 cm, and the fibers were collected on aluminum foil or baking paper on the collector. Each formulation was loaded into a 5 mL polypropylene syringe purchased from B.Braun SE, Germany. For monoaxial electrospinning, the formulations were pumped through a VIEWEG GmbH (Germany) dosing needle with an inner diameter of 0.41 mm and a capillary length of 12.7 mm. For coaxial electrospinning, the formulations were pumped through a VIEWEG GmbH (Germany) dosing needle with an inner diameter of 1.19 mm. The inner needle of the coaxial electrospinning head (BIOINICIA FLUIDNATEK SLU, Spain) had an inner diameter of 0.6 mm and an outer diameter of 0.9 mm. The flow rate for the integrated syringe pump was between 600 μL/h and 1200 μL/h in the case of monoaxial electrospinning. For the coaxial electrospinning, the flow rate of the core phase was 600 μL/h, and for the shell phase, 110 μL/h. The crosslinker concentrations of the electrospinning formulations and the molar ratios of reactive VECOLLAN® groups to reactive crosslinker groups are listed in Table 1.

**Table 1:** Composition and crosslinker concentration of various formulations.

Formulation name	Crosslinker concentration in shell phase [mg/mL]	Molar ratio (CLP: Crosslinker)
CLP:DMTMM 1:0.6	173.2	1:0.6
CLP:DMTMM 1:0.1	28.9	1:0.1
CLP:DMTMM 1:0.08	23.1	1:0.08
CLP:DMTMM 1:0.04	11.5	1:0.04
CLP:PEG 4-Arm CL 1:0.1	164.8	1:0.1
Control (no crosslinker)	/	/

### 2.4 Calculation of the molar ratio between VECOLLAN® and crosslinker

For DMTMM, the ratio of reactive groups of VECOLLAN® to a molecule of crosslinker refers to the number of carboxylic acid groups of one molecule of VECOLLAN®. One molecule of DMTMM has one reactive site that can undergo crosslinking reactions. Therefore, a ratio of reactive groups to DMTMM of 1:1 corresponds to one molecule of VECOLLAN® to 36 molecules of DMTMM.

For the 4-Arm-polyethylene glycol-succinimidyl-glutarate-ester (PEG 4-Arm CL), the ratio of reactive groups of VECOLLAN® to a molecule of crosslinker refers to the number of primary amines of one molecule of VECOLLAN®. One molecule of PEG 4-Arm CL has four reactive sites that can undergo crosslinking reactions. Therefore, a ratio of reactive CLP groups to PEG 4-Arm CL of 1:1 corresponds to one molecule of CLP to 5.75 molecules of PEG 4-Arm CL.

### 2.5 Prepared formulations

See Table 1 here.

### 2.6 Scanning electron microscopy

Before scanning electron microscope (SEM) analysis, the fibers were Au/Pd sputtered in a Leica EM SCD500 sputter coater (Leica Microsystems GmbH, Germany) for 60 s in an argon phase. The surface of the samples was examined using a JSM-IT 300 scanning electron microscope (JEOL Ltd., Japan). The microscope was operated at an acceleration voltage of 10 kV.

#### 2.6.1 SEM pictures of samples in “wet state”

A drop of water was applied to the sample and left to act for 2 h. Afterwards, the sample was freeze-dried. The freeze-drying process was conducted using a Martin Christ GmbH, Epsilon freeze-drying system (Osterode, Germany). The fibers were loaded into the chamber, and the vacuum was applied to achieve approximately 100 mTorr while maintaining a temperature of around 0 °C for 30 min. The temperature was then gradually decreased to −40 °C over the next four hours. Following this, the temperature was raised to −10 °C while maintaining low pressure for 36 h. Finally, the temperature was increased to 20 °C for an additional two hours. After the process, the vacuum was released, and the freeze-dried samples were investigated via SEM.

### 2.7 Transmission electron microscopy

For the transmission electron microscopy (TEM) analysis, the fibers were embedded in an epoxy resin. The fibers in the epoxy resin were cut in ultrathin sections, approximately 160 nm thick, using a Leica UC7 ultramicrotome (Leica Microsystems GmbH, Germany). These sections were subsequently placed onto carbon-coated copper grids to maintain stability during imaging. Fibers examined in the side view in the TEM were deposited directly in the electrospinning process on the carbon-coated copper grid.

TEM investigations were conducted using a JEOL JEM-2100Plus transmission electron microscope (JEOL Ltd., Japan) operated at an accelerating voltage of 200 kV.

### 2.8 Dissolution of nonwoven mats

The dissolution tests were conducted by adding a nonwoven mat (10 mg of VECOLLAN® equivalent) to a 30 mL glass bottle with a lid. 10 mL of MQ water were added, and (100 μL) samples were collected from the media at 1 h, 3 h, and 24 h intervals. The samples were collected in a 2 mL Eppendorf safe lock tube (Eppendorf SE, Germany) each. The samples were centrifuged in an Eppendorf 5430 R centrifuge at 3000 RPM for 3 min to prevent possible nonwoven mat fragments from being analyzed as well. After that, equivalents of the supernatant were analyzed in triplicate per Bicinchoninic Acid Assay (Pierce BCA Protein Assay, Thermo Fisher Scientific, USA) to determine the VECOLLAN® concentration in solution.

#### 2.8.1 Preparation of standards and calibration curve

A stock solution of VECOLLAN® (10 mg/mL) was prepared in double-distilled water. Calibration curves (16 μg/mL to 2000 μg/mL) were prepared by subsequent dilution in double-distilled water. The BCA working reagent was prepared by mixing 50 parts of Reagent A with 1 part of Reagent B, maintaining a 50:1 ratio. For the assay procedure, 25 μL of each standard was pipetted into the designated wells of a 96-well microplate. Subsequently, 200 μL of the BCA working reagent was added to each well and mixed thoroughly. The plate was then covered and incubated at 37 °C for 30 min.

#### 2.8.2 Absorption measurement and quantification

Absorption measurement was performed with an Infinite 200 Pro multiplate reader (Tecan Trading AG, Switzerland). The absorbance of the samples was measured in a 96-well microplate (Greiner AG, Austria) at 562 nm after the same treatment as the standard curve dilutions described in 2.8.1. The protein concentration was determined by interpolating the sample absorbance values on the standard curve.

### 2.9 Fiber and bead diameter measurement

ImageJ software (National Institutes of Health, USA) was used to measure fiber diameters based on the SEM images. 100 different positions on each sample were measured for the fibers, respectively, the fiber-like portions of the beaded fibers. For the beads, 20 positions per sample were measured.

### 2.10 Viscosity measurements

The viscosity measurements were conducted using an Anton-Paar MCR 502 WESP system equipped with a plate-cone extension. The lower plate was flat (stainless steel, Ø 50 mm, CP50) while the upper cone had a 1° angle (stainless steel, Ø 50 mm, CP50–1) with a truncation of 99 μm. A sample load of 750 μL was applied. Excess sample was removed with a tissue after reaching the measurement position (0.099 mm measurement gap). The shear rate was measured at a constant 21 ± 1 °C with 20 data points between 1 1/s and 500 1/s as a logarithmic ramp. For the measurement value, the average viscosity [mPa*s] of measurement point 4 (79.8 1/s) to measurement point 9 (211 1/s) was calculated.

### 2.11 Mechanical properties

The mechanical properties, namely the “tensile strength at maximum load” and “elongation at break”, of the nonwoven mat composed of electrospun cross-linked VECOLLAN®/PEO fibers were evaluated. The samples, which were strips cut from the baking paper used to collect the fibers, measured 5.3 × 20 mm (*n* = 5). Testing was conducted using a static mechanical analyzer (INSTRON 3366, Instron Deutschland GmbH, Germany) at 21 ± 1 °C. The tensile rate was set at 10 mm/min, beginning at an initial load of 0.01 N.

The thickness of each sample strip was determined using a laser profilometer (TalyScan 150, Taylor Hobson), and the “apparent cross-section area” was subsequently calculated. The measurement values were directly obtained from the Instron software.

## 3 Results and discussion

### 3.1 Generating VECOLLAN® fibers

To produce fibers from a material by electrospinning, the molecular weight is, in addition to other variables, an important factor (Al-Abduljabbar and Farooq, 2022; Xue et al., 2019). With a molecular weight of 22.8 kDa, VECOLLAN® could not generate fibers from a pure VECOLLAN® electrospinning formulation. Next, we tested generating fibers from VECOLLAN® by partially crosslinking the molecules with small amounts of crosslinker. This should increase the average molecular weight and thus enable the molecules to entangle with the now longer molecules on average. Thus, it was important to choose a crosslinker quantity that is so small that no complete hydrogel is formed. If a hydrogel forms before the spin formulation is processed in the electrospinning equipment, further processing would no longer be possible. However, this approach also failed.

Since fiber generation with a pure VECOLLAN® formulation did not work, we combined it with a high molecular weight polyethylene oxide (PEO). The principle of combining a low molecular weight molecule with a high molecular weight molecule to enable fiber generation is also known in the literature (Bonino et al., 2011).

**Fig. 1:**
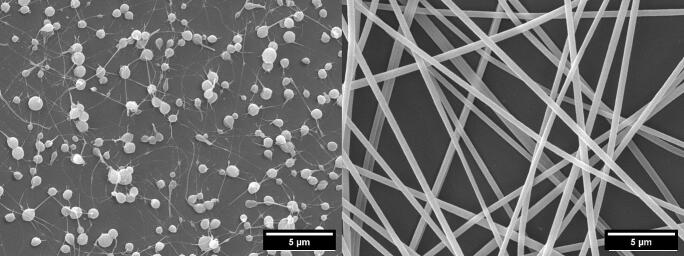
Impact of polymer formulation composition on the morphology of electrospun fibers. Left: 20 mg/mL VECOLLAN®, 11.2 mg/mL PEO. Right: 120 mg/mL VECOLLAN®, 18.6 mg/mL PEO.

Polyethylene oxide is an established, well-electrospinnable polymer (Schneider et al. (2016); Son et al. (2004)) with FDA approval in the biomedical sector (Kalalinia et al., 2021). The PEO serves as a supporting structure for the VECOLLAN® molecules, thus enabling the formation of fibers. Fig. 1 (left) shows the first fibers that could be generated by combining VECOLLAN® with PEO. The fibers show many large beads and only thin fibrous connections between the beads. By increasing the VECOLLAN® and PEO concentration of the formulation, uniform VECOLLAN® fibers could be generated, which consist of approximately 86 % (*w*/w) VECOLLAN® and 14 % (w/w) PEO (Figure 1 right). The optimized VECOLLAN®/PEO electrospinning formulation consists of 120 mg/mL VECOLLAN® and 18.6 mg/mL of PEO in a mixture of double-distilled water and acetone [9:1 (*v*/v)].

The generated VECOLLAN®/PEO fibers are water-soluble and dissolve within a short time. To ensure that these fibers are suitable for biomedical applications and have a longer lifespan, cross-linking is essential. There are various ways described in the literature to crosslink nanofibers.

Cao et al. (2010) crosslinked nanofibers by placing them in a solution containing a crosslinking agent after electrospinning. The disadvantage of this method is that it is a two-step process. In the first step, the water-soluble fibers are generated. In the second step, these water-soluble fibers are transferred into a crosslinker-containing solution.

Meng et al. (2012) crosslinked electrospun fibers by blending the crosslinker and the components to be crosslinked before starting the electrospinning process. The advantage of this method compared to the previously described two-step method is that an additional step is avoided. However, the disadvantage is that there is a limited time frame in which the electrospinning formulation can be electrospun, since the crosslinking process already takes place in the bulk vessel. Another disadvantage of this method is that the viscosity of the formulation increases during the electrospinning process. This increase in viscosity leads to a change in the fiber morphology. (Tiwari and Venkatraman, 2012b). Due to the disadvantages of the two crosslinking methods described by Cao et al. (2010) and Tiwari and Venkatraman (2012a), we decided to use a coaxial crosslinking approach.

**Fig. 2:**
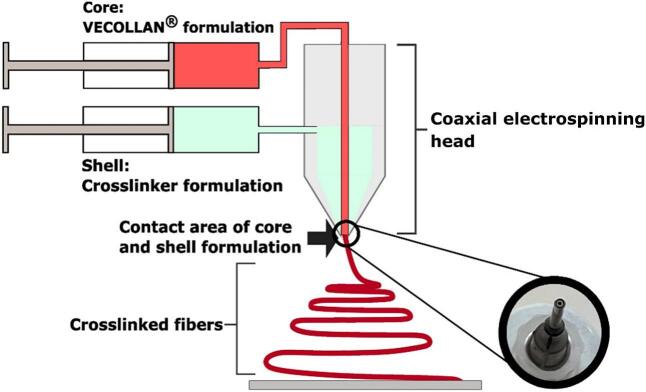
Schematic illustration of the coaxial crosslinking electrospinning process.

The coaxial crosslinking approach involves using a coaxial electrospinning head with a nozzle inside the nozzle (Fig. 2). The VECOLLAN®/PEO electrospinning formulation is pumped in the inner nozzle, while the crosslinker-containing solution is fed through the outer nozzle. To generate water-insoluble fibers, a sufficient concentration of crosslinker has to be fed through the outer nozzle, concerning the substance to be crosslinked in the inner nozzle. Coaxial crosslinking is a one-step method in which the crosslinker and VECOLLAN® are combined in situ during electrospinning. By combining them during the electrospinning process, there is no limited time frame in which water-insoluble fibers can be generated. Since there is no limited time frame and coaxial crosslinking is a one-step method in which the formulation can be processed, this method is also interesting for scale-up. A challenge observed with coaxial crosslinking is that it is difficult to generate bead-free fibers.

The optimized electrospinning formulation was used for all formulations in Table 1. Electrospinning was conducted in a coaxial electrospinning head. The nozzles of the electrospinning head end at the same height (Fig. 2).

The crosslinker type and concentration of the shell phase in the coaxial electrospinning process were varied. The molar ratios between the reactive groups of VECOLLAN® and the crosslinker were calculated based on the concentration of the crosslinker solution and the flow rates of the core and shell solutions. A constant electrospinning process was possible for all formulations.

### 3.2 Dissolution of fibers

**Fig. 3:**
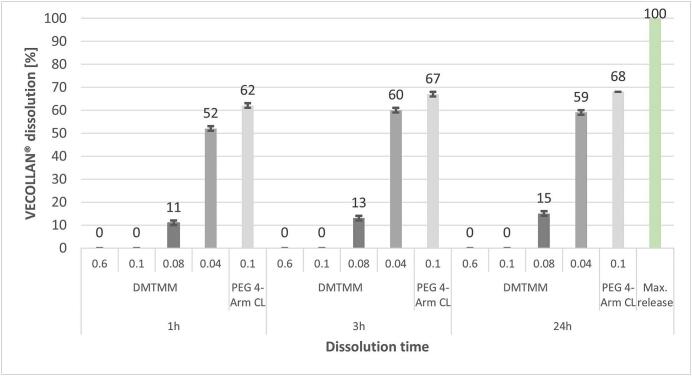
Influence of incubation time and crosslinking conditions on the amount of VECOLLAN® released from electrospun fibers after 1, 3, and 24 h in water. 100 % dissolution corresponds to 1 mg/mL VECOLLAN® in the dissolution medium.

Fig. 3 shows the VECOLLAN® dissolution of the VECOLLAN®/PEO fibers in double-distilled water. The VECOLLAN® concentration in the dissolution medium was analyzed after 1 h, 3 h, and 24 h. The dissolution speed of VECOLLAN® depended on the kind of crosslinker and its concentration. The maximum release for each sample was 1 mg/mL. Control samples without crosslinker were completely dissolved after 1 h. DMTMM in a molar ratio of 1:0.6 and 1:0.1 led to complete VECOLLAN® crosslinking and no detectable dissolution after 24 h. Interestingly, a partial VECOLLAN® release could be observed in the DMTMM MR 1:0.08 and 1:0.04 cases. The amount of VECOLLAN® released can therefore be controlled depending on the amount of crosslinker. This control of release can be interesting for several reasons, for example, for wound dressings where the wound-healing properties of the VECOLLAN® could take effect through release. The fibers with PEG 4-Arm CL in the molar ratio of 1:0.1 showed partial VECOLLAN® dissolution.

The comparison of the DMTMM crosslinker and the PEG 4-Arm CL shows that the VECOLLAN® release depends not only on the molar ratio between VECOLLAN® and the crosslinker used but also on the type of crosslinker.

### 3.3 Fiber characteristics before and after the addition of water

#### 3.3.1 Fiber morphology

**Fig. 4:**
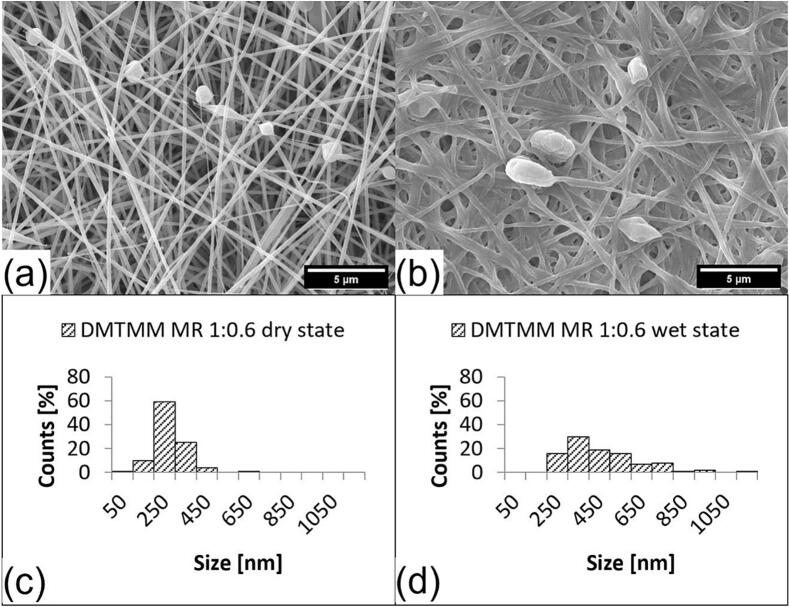
SEM pictures and fiber size distribution of crosslinked VECOLLAN®/PEO fibers combined with DMTMM (Molar ratio 1:0.6). (a) Dry state, (b) Wet state, (c) Average diameter: 273.0 nm, Standard deviation: 76.1 nm, Median: 268.4 nm, (d): Average diameter: 459.1 nm, Standard deviation: 180.2 nm, Median: 424 nm.

**Fig. 5:**
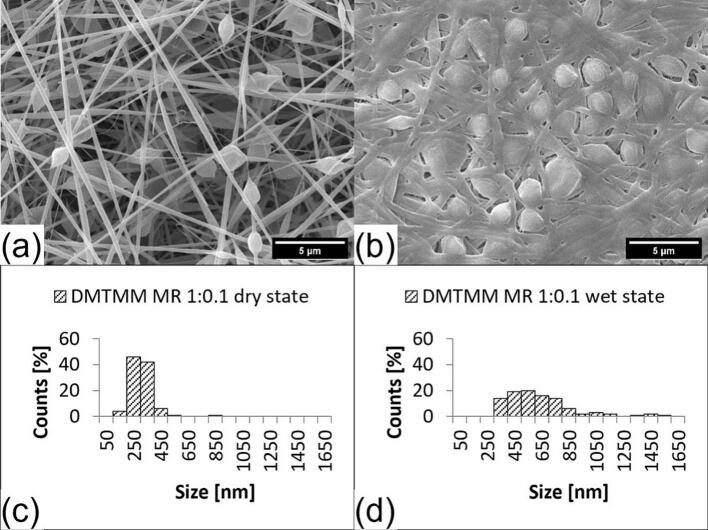
SEM pictures and fiber size distribution of crosslinked VECOLLAN®/PEO fibers combined with DMTMM Molar ratio 1:0.1). (a) Dry state, (b) Wet state, (c): Average diameter: 307.5 nm, Standard deviation: 88.8 nm, Median: 301.0 nm, (d): Average diameter: 631.5 nm, Standard deviation: 249.2 nm, Median: 583 nm.

**Fig. 6:**
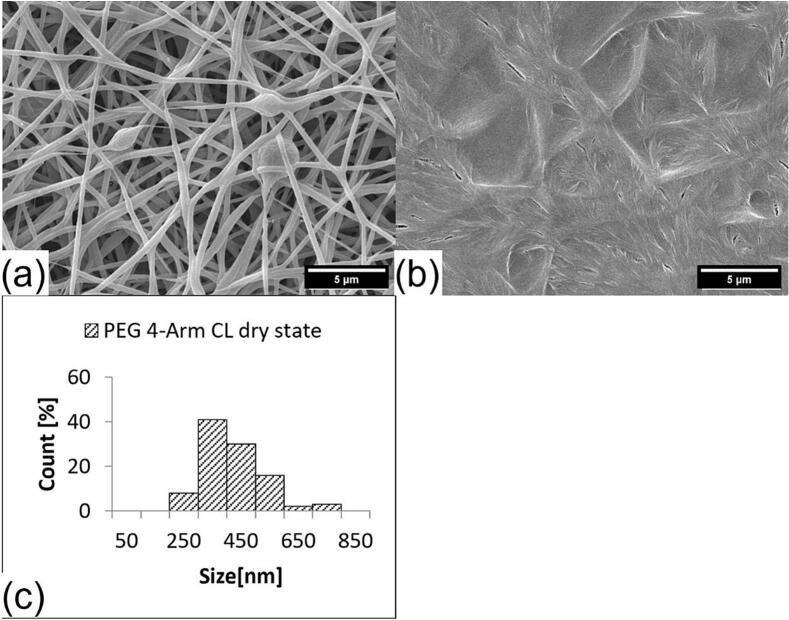
SEM pictures and fiber size distribution of crosslinked VECOLLAN®/PEO fibers combined with PEG 4-Arm CL (Molar ratio 1:0.1). (a) Dry state, (b) Wet state, (c) Average diameter: 420.0 nm, Standard deviation: 98.2 nm, Median: 401.3 nm. No measurement was possible for the wet state.

The fiber morphology was investigated using scanning electron microscopy (SEM). SEM pictures were taken before (dry state) and after the addition of water (wet state). Depending on the solution that was fed through the outer nozzle in the coaxial electrospinning head, the fibers show more or fewer beads (Fig. 4, Fig. 5, Fig. 6 and S1 to S3, see supplementary material). Since all electrospinning parameters were kept constant, the differences in fiber morphology could be caused by the difference in the electrical conductivity of the solution that is fed through the outer nozzle. In general, an increasing concentration of ions in a formulation increases the electrical conductivity (Angammana and Jayaram, 2011).

The fiber diameter was determined using the ImageJ software. However, this information about the diameter increase only considers the increase of one dimension. To determine the increase in volume in three dimensions due to the addition of water, the following assumptions are made:

1. The fibers have the shape of an ideal cylinder.
2. The width swelling corresponds to the length swelling.
3. The beads are perfectly spherical.

For the calculation of the increase in diameter and volume, the median diameters before and after the addition of water were compared, since the weighting of particularly thick or particularly thin fibers is lower. This is particularly useful for the fibers after the addition of water, since here, probably several fibers often merge into one fiber, and therefore, it can no longer be said that this is an increase in the diameter of a single fiber.

Changes in the bead and fiber morphology were evaluated after the addition of water. Whereas no correlation between bead diameter increase and the cross-linker molar ratio could be observed (Table 2), a clear trend in the fiber diameter changes could be observed for the fibers and the fiber-like portions of the beaded fibers. Fig. 4, Fig. 5 and supplementary material S1 show the diameter of fibers with DMTMM MR of 1:0.6, 1:0.1, and 1:0.08. The median increases from the dry to wet state are 58.1 %, 94 % and 88.6 %, respectively. The weaker swelling of the sample with DMTMM MR 1:0.08, compared to the one with DMTMM MR 1:0.1, could be explained by the fact that a small amount of VECOLLAN® could have been released from the fibers, which was not the case with the DMTMM MR 1:0.1 sample. Generally speaking, adding water increases the maximum fiber diameter, and the diameter distribution becomes wider for the samples that kept the fiber structure in the wet state.

**Table 2:** Bead size, bead frequency, and volume calculation of beads and fibers for VECOLLAN®/PEO nanofibers in the dry and wet state.

Sample	Median bead diameter [nm]		Factor fiber volume increase	Amount of beads	Factor bead volume increase
	Dry state	Wet state			
DMTMM MR 1:0.6	1189.0	1786.6	3.95	some	3.39
DMTMM MR 1:0.1	1313.1	1652.1	5.93	more	1.7
DMTMM MR 1:0.08	1984.1	3116.4	5.62	more	1.89
DMTMM MR 1:0.04	1413.7	/	/	more	/
PEG CL MR 1:0.1	1717.1	/	/	some	/
Control	1194.9	/	/	more	/
DMTMM MR 1:0.1 + HA 0.4 %	/	/	7.89	few	/
DMTMM MR 1:0.1 + HA 0.6 %	/	/	6.42	few	/

Fig. 4 displays the VECOLLAN®/PEO fibers with a DMTMM molar ratio of 1:0.6. The fibers show a uniform appearance and some beads. After the addition of water, the median fiber diameter increased by 58.1 %. The diameter increase of the beads is shown in Table 2. For the beads, no regularity or correlation regarding diameter and diameter increase was found between the different molar ratios of crosslinker and type of crosslinker. In contrast, the median diameter increases by 94 %, measured for the fibers with DMTMM MR 1:0.1 (Fig. 5). The fibers with DMTMM MR 1:0.08 show a diameter increase of 88.6 % from dry to wet state (see supplementary material, Fig. S1). Interestingly, a trend towards greater swelling of the fibers in water at a lower DMTMM molar ratio can be observed. A stronger swelling at a lower crosslinker concentration is expected because there are fewer intermolecular bonds within the fiber, and the water can penetrate better. A comparable effect has been described for PVA membranes that were cross-linked with glutaraldehyde in various concentrations (Kwang-Je et al., 1993).

The fibers that were produced with DMTMM in a MR of 1:0.04 (see supplementary material, Fig. S2) lose their structure to a large extent after the addition of water, and a reliable measurement of the fiber thicknesses was not possible. This strong loss of structure was to be expected since the fibers with DMTMM MR 1:0.04 showed a high VECOLLAN® release of 52 % after 1 h in the dissolution test (Fig. 3). The fibers containing the PEG 4-Arm CL could not be measured in the wet state. This observation also corresponds to the result of the dissolution test (Fig. 3), since after 1 h, 62 % of VECOLLAN® was released. For the control fibers (see supplementary material, Fig. S3) without a crosslinker, no fiber diameter could be determined in the wet state due to the loss of structure.

The analysis of variance (ANOVA; Tukey, 95 % Confidence interval) of 100 fiber diameters measured of each sample revealed that that in the dry state, the diameters of the fibers of the PEG CL MR 1:0.1 sample were significantly higher than the fiber diameters of the other samples in the dry state, while the fiber diameters of the remaining samples (DMTMM MR 1:0.6, DMTMM MR 1:0.1, DMTMM MR 1:0.08 and DMTMM MR 1:0.04) did not differ from one another in the dry state. The fiber diameters of the measured fibers in the wet state differ significantly from their fiber diameters in the dry state. This aligns with expectations, as a marked increase in fiber diameter from the dry to the wet state was observed for all fibers. Additionally, the fiber diameters of the samples DMTMM MR 1:0.6, DMTMM MR 1:0.1, and DMTMM MR 1:0.08 also differ significantly from one another.

#### 3.3.2 Swelling behavior when adding hyaluronic acid to VECOLLAN®/PEO formulations

Hyaluronic acid (HA) is a naturally occurring glycosaminoglycan widely distributed throughout connective, epithelial, and neural tissues. It is known for its exceptional capacity to retain water, with the ability to bind up to 1.000 times its weight in water, making it an important component in maintaining skin hydration and elasticity (Stern et al., 2006). HA is also known for its role in tissue repair and its anti-inflammatory properties, which contribute to its use in various medical and cosmetic applications (Fallacara et al., 2018).

Due to its good usability for medical and cosmetic applications, it was tested as a component in the VECOLLAN®/PEO electrospinning formulation. On the one hand, it was examined whether the swelling of the fibers could be controlled by adding a very hygroscopic material. On the other hand, the influence of an increase in viscosity on the fiber morphology was investigated.

Two different formulations with different amounts of HA were prepared. One formulation with 0.4 % (*w*/w) HA and one with 0.6 % (w/w) regarding the total weight of the electrospinning formulation. The formulations were processed separately in the electrospinning equipment. Furthermore, they were combined with DMTMM via coaxial crosslinking and a molar ratio of 1:0.1 regarding the reactive groups of VECOLLAN®.

**Fig. 7:**
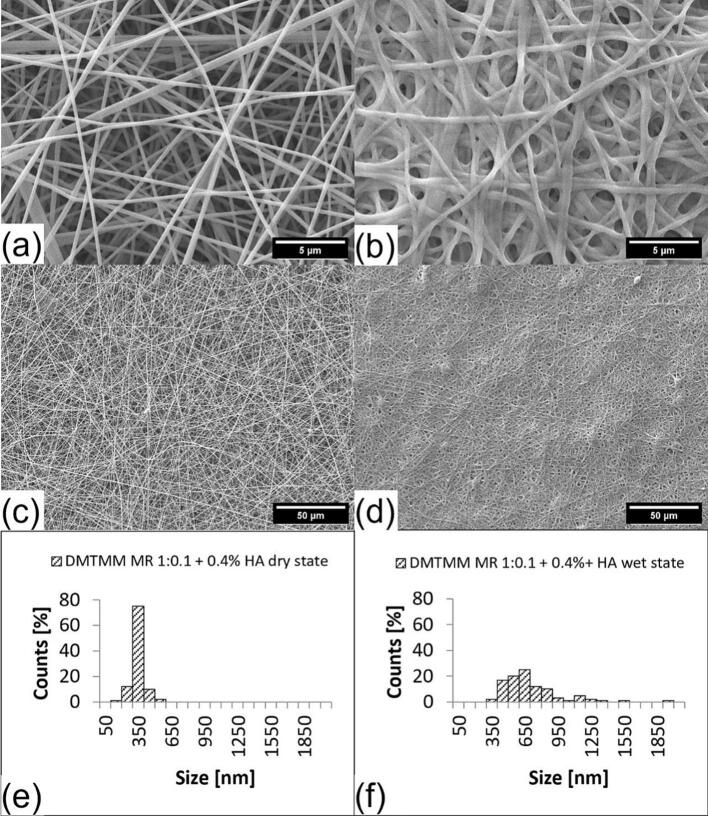
SEM pictures and fiber size distribution of VECOLLAN®/PEO + 0.4 % (w/w) HA and DMTMM (Molar ratio 1:0.1). (a) Dry state, (b) Wet state, (c) dry state overview, (d) wet state overview, (e) Average diameter: 291.1 nm, Standard deviation: 55.9 nm, Median: 293.4 nm, (f) Average diameter: 655.1 nm, Standard deviation: 257.3 nm, Median: 590.2 nm.

The fiber diameter of the 0.4 % HA formulation increased by 101.2 % and of the 0.6 % HA formulation by 101.6 % from dry to wet state. This fiber diameter increase is, as expected, significant, while there was no difference between the two samples in the wet state (ANOVA analysis, Tukey, 95 % Confidence interval). Compared to the formulation without HA, the fibers showed a slightly greater degree of swelling after the addition of water (Table 2). However, the change in fiber morphology (Fig. 7) and the increase in viscosity of the electrospinning formulation (Table 3) were even more noticeable.

**Table 3:** Viscosity measurements of VECOLLAN®/PEO electrospinning formulations with and without hyaluronic acid addition.

Sample	Viscosity [mPa*s]
VECOLLAN®/PEO formulation	1224
VECOLLAN®/PEO formulation + 0.4 % (w/w) Hyaluronic acid	1974
VECOLLAN®/PEO formulation + 0.6 % (w/w) Hyaluronic acid	2723

**Fig. 8:**
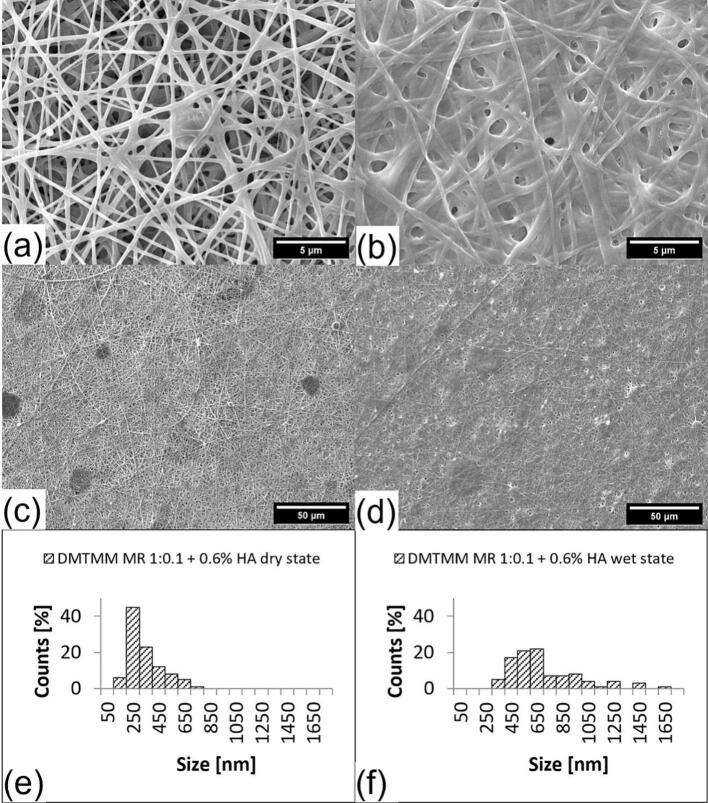
SEM pictures and fiber size distribution of VECOLLAN®/PEO + 0.6 % (w/w) HA and DMTMM (Molar ratio 1:0.1). (a) Dry state, (b) Wet state, (c) dry state overview, (d) wet state overview, (e) Average diameter: 341.2 nm, Standard deviation: 131.2 nm, Median: 294.2 nm, (f) Average diameter: 654.6 nm, Standard deviation: 274.9 nm, Median: 576.2 nm.

Due to the strong viscosity increase of the electrospinning formulation, caused by hyaluronic acid (Table 3), an increase in voltage (HV+) to 22 kV had to be conducted to enable the formation of the tailor cone and electrospinning. As can be seen in Fig. 7, for the VECOLLAN®/PEO + 0.4 % (*w*/w) HA formulation, constant electrospinning without interruption or formulation droplets that landed on the collector was possible. Constant electrospinning was impossible for the VECOLLAN®/PEO + 0.6 % (w/w) HA formulation under the same conditions (Fig. 8). The Taylor cone kept coming off, and there were drops of the formulation on the produced fibers (Fig. 8 (c)). According to the literature, the combination of increased viscosity of the electrospinning formulation and applying increased voltage in the electrospinning process promotes the formation of bead-free fibers (Fong et al., 1999). As can be seen in Fig. 7 (a) and Fig. 7 (c), this principle of increased viscosity and increased voltage for bead reduction also worked in the case of the VECOLLAN®/PEO + HA fibers.

### 3.4 Fibers loaded with silver nanoparticles

Silver nanoparticles were used to investigate their distribution within the fiber through the coaxial electrospinning process. Since the silver nanoparticles are only 5 nm in size and a crosslinker molecule like DMTMM has a similar dimension (Clayden et al., 2012), this experiment suggests a potential distribution of the crosslinker within the fiber, although no definitive statement can be made, since crosslinker and silver nanoparticles are not linked. In addition to being easily visible under a transmission electron microscope due to their high density, silver nanoparticles also have antibacterial properties (López-Esparza et al., 2016). One potential application of these silver particle-loaded fibers is the prevention of postoperative infections. However, this study did not examine whether the fibers have an antibacterial effect.

**Fig. 9:**
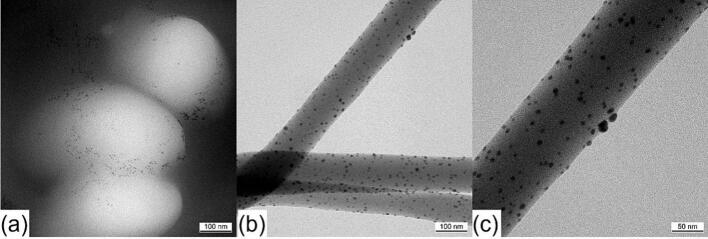
Transmission electron microscope pictures of VECOLLAN®/PEO + Ag 2200 ppm + DMTMM 0.1. (a) Nanofiber cross-section, (b) + (c) Nanofiber side view.

**Fig. 10:**
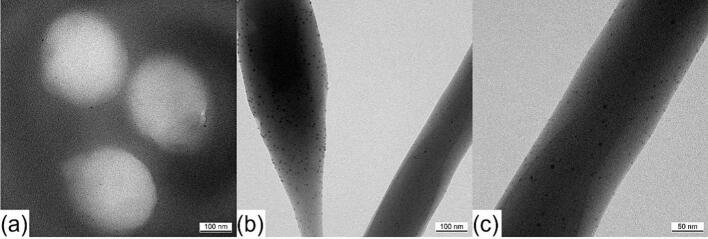
Transmission electron microscope pictures of VECOLLAN®/PEO + Ag 2200 ppm without crosslinker. (a) Nanofiber cross-section, (b) + (c) Nanofiber side view.

The sample in Fig. 9 consists of VECOLLAN®/PEO fibers that contain DMTMM in MR 1:0.1. Instead of dissolving DMTMM in double-distilled water, it was dissolved in an aqueous 2200 ppm silver nanoparticle suspension. This suspension, containing the DMTMM, was used in the shell phase of the coaxial electrospinning head to crosslink VECOLLAN® in the core phase. The second sample (Fig. 10) contains no DMTMM. For this sample, the 2200 ppm silver nanoparticle suspension was pumped into the shell phase of the coaxial electrospinning head. The composition of both prepared samples is shown in Table 4

**Table 4:** Samples containing silver (Ag) nanoparticles investigated in the transmission electron microscope (TEM).

Sample	Ag-Suspension concentration [ppm]	DMTMM [Molar ratio]
VECOLLAN®/PEO + Ag 2200 + DMTMM 0.1	2200	0.1
VECOLLAN®/PEO + Ag 2200 + NO DMTMM	2200	/

Fig. 9 (a) shows the cross-section of three nanofibers containing DMTMM and silver nanoparticles. The silver nanoparticles seem to be mainly located in the outer area of the fibers. Nevertheless, various experiments have shown that the coaxial crosslinking of the fiber can be sufficient to crosslink VECOLLAN® and obviate dissolution in a dissolution test. A side view of these fibers is shown in Fig. 9 (b) and (c). The figures illustrate the uniform distribution of silver nanoparticles along the entire length of the fibers. This uniform distribution along the fibers was surprising since the flow rate of the core phase (600 μL/h) is 5.45 times higher than that of the shell phase (110 μL/h). A comparable distribution of silver nanoparticles is shown by the fibers in which no crosslinker was added in the shell phase (Fig. 10).

### 3.5 Mechanical properties

The mechanical properties of electrospun nanofibrous scaffolds are crucial for their application in biomedical fields, as they significantly affect in vitro cell behaviors such as migration, proliferation, differentiation, and morphology. Therefore, understanding the mechanical properties of the fibers determines their area of use (Croisier et al., 2012). To assess the mechanical properties of the fibers, measurements were carried out with a static mechanical analyzer (Instron 3366). The tensile strength and the elongation at break were determined.

**Fig. 11:**
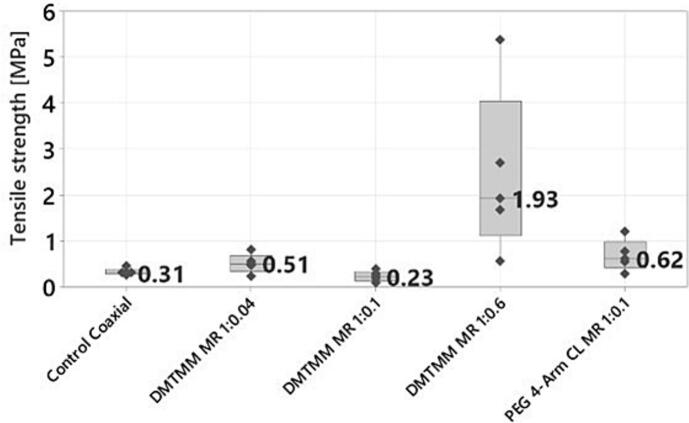
Tensile stress at a maximum of coaxially produced nonwoven mats. Dark grey dots show measurement values. The number represents the median value.

For the coaxially produced samples, the DMTMM MR 1:0.6 sample showed the highest tensile strength (Fig. 11). The ANOVA analysis (Tukey, 95 % Confidence interval) revealed that DMTMM MR 1:0.6 withstands significantly higher tensile strength than the other groups, while no significant differences were observed among the other samples. Small amounts of crosslinker (DMTMM MR 1:0.04, DMTMM MR 1:0.1, and PEG4-Arm CL MR 1:0.1) do not appear to be sufficient to significantly increase the tensile strength compared to the control. The DMTMM MR 1:0.6 sample shows the highest tensile strength, which is to be expected since most of the intermolecular bonds are present here, which makes the fleece more stable. There was also an indication of an influence concerning the type of crosslinker. The PEG 4-Arm CL MR 1:0.1 sample shows a slightly higher tensile strength compared to DMTMM MR 1:0.1 and DMTMM MR 1:0.04. The sample DMTMM MR 1:0.04 shows higher tensile strength than DMTMM MR1:0.1. Since not only the amount of crosslinker but also the structure and thickness of the fibers affect their tensile strength. One possible explanation could be that thinner fibers are packed more densely, which results in fewer voids among the fibers. This could increase the tensile strength of the fleece. A similar observation was described by Huang (Huang et al., 2004), where the finest fiber mat of different gelatin fiber mats was the most stable.

**Fig. 12:**
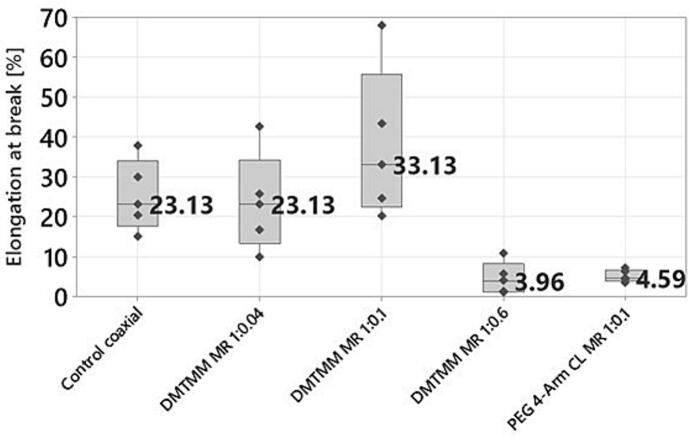
Elongation at break of coaxially produced nonwoven mats. Dark grey dots show measurement values. The number represents the median value.

In Fig. 12, the elongation at break of the coaxially produced samples is presented. The ANOVA analysis reveals a significant difference between the DMTMM 0.1 samples (highest elongation at break) and the DMTMM MR 1:0.6 and PEG 4-Arm CL MR 1:0.1 (lowest elongation at break). It was to be expected that DMTMM MR 1:0.6 would show the lowest elongation at break, since it has the highest crosslinker concentration and thus the most intermolecular bonds, which make the nonwoven mat stiffer. Although the molar ratio for DMTMM MR 1:0.1 and PEG 4-Arm CL 1:0.1 refers to different reactive groups of VECOLLAN®, the added crosslinker amounts are in a similar range. The strong difference shows that PEG 4-Arm CL produces stiffer nonwoven mats. Thus, in addition to the amount of crosslinker, the stiffness of the nonwoven mat also depends strongly on the type of crosslinker.

**Fig. 13:**
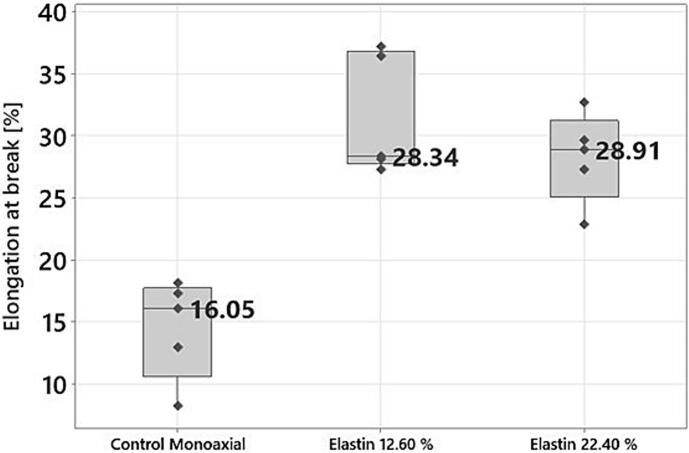
Elongation at break of monoaxially produced nonwoven mats without crosslinker. Dark grey dots show measurement values. The number represents the median value.

For the monoaxially produced samples, non-crosslinked VECOLLAN®/PEO fibers were compared with fibers that contain additional elastin (Fig. 13). The sample named “Elastin 12.60 %” consists of 12.60 % (*w*/w) elastin, and the sample named “Elastin 22.40 %” consists of 22.40 % (w/w) elastin. Elastin is a key protein that provides essential stretch and flexibility in the extracellular matrix (ECM) present in various tissues, including skin, lungs, arteries, ligaments, and cartilage (Del Prado-Audelo et al., 2020; Kim and Chaikof, 2010). The experiment aims to find out whether the stretch or flexibility can be increased by an additive such as elastin.

On the one hand, ANOVA analysis (Tukey, 95 % Confidence interval) reveals that there is no significant difference in tensile strength between control and elastin-containing samples. This result meets the expectation since the samples do not contain a crosslinker. On the other hand, it was found that the samples containing elastin had a significantly greater elongation at break than the control sample without elastin. This was to be expected since elastin is known for its stretch and flexibility and fulfills this function in many tissues in the human body. This means that, depending on the application area of such fibers, the elongation at break could also be adapted to the required needs. No significant difference in elongation at break could be determined between the two elastin samples. The differences in elongation at break between the control coaxial (Fig. 12) and the control monoaxial (Fig. 13) can be explained by the fact that the needles of the co- and monoaxial electrospinning heads can influence the fiber morphology and fiber thickness (Gündüz, 2023). Since these factors also influence the mechanical properties of electrospun fibers, this could explain the differences (Huang et al., 2004).

## 4 Conclusion

In this study, we successfully generated VECOLLAN®-based fibers that exhibit varying degrees of mechanical stability and dissolution kinetics, influenced by the concentration and amount of crosslinker used. Fibers with DMTMM at a molar ratio of 1:0.1 showed no dissolution of VECOLLAN® after 24 h, establishing the lower limit of complete crosslinking. The fibers demonstrated distinct swelling behaviors upon water exposure, indicating different capacities for water absorption. The fibers with the highest DMTMM MR of 1:0.6 exhibit weaker swelling upon water exposure. The volume increase factor was 3.95 for DMTMM 1:0.6 and 5.93 for DMTMM 0.1, highlighting the adaptable water absorption capacity of the fibers depending on the crosslinker concentration. The production of uniform fibers was challenging, but this issue was solved by adding hyaluronic acid, which increased the viscosity of the formulation. By adjusting the voltage, we were able to generate uniform fibers. Notably, incorporating hyaluronic acid has further enhanced the swelling properties, suggesting potential applications in the wound healing sector.

Additionally, we investigated the silver nanoparticle distribution within and along the fibers, which indicates the homogeneity of the coaxial electropspinning process. The mechanical properties of the fibers were also thoroughly investigated; the fibers with DMTMM MR 1:0.6 were the most stable and rigid, while the addition of elastin to non-crosslinked fibers significantly improved their elongation at break.

The findings presented in this paper provide a solid foundation for the development of electrospun fibers composed of the non-animal-derived collagen-like protein VECOLLAN®. Furthermore, to the best of our knowledge, this study represents the first demonstration of a coaxial crosslinking process utilizing the described setup. Future experiments should focus on assessing the biocompatibility of these fibers with cells, which will be crucial for advancing their potential applications as drug carriers or cell scaffolds. Overall, our results underscore the promising properties of VECOLLAN®-based fibers for biomedical applications, paving the way for innovative solutions in drug delivery and the medical device sector.

## CRediT authorship contribution statement

**Krauss Christoph:** Writing – original draft, Validation, Methodology, Investigation, Formal analysis, Data curation, Conceptualization. **Montero Mirabet Maria:** Writing – review & editing, Supervision, Resources, Project administration, Conceptualization. **Zhang Jian-Feng:** Writing – review & editing, Methodology, Investigation, Formal analysis, Data curation. **Mäder Karsten:** Writing – review & editing, Supervision, Conceptualization.

## Declaration of competing interest

The authors declare the following financial interests/personal relationships which may be considered as potential competing interests:

Christoph Krauss reports financial support was provided by Evonik Industries AG. Maria Montero Mirabet reports financial support was provided by Evonik Industries AG. Jian-Feng Zhang reports financial support was provided by Evonik Industries AG. Christoph Krauss reports a relationship with Evonik Industries AG that includes: employment and equity or stocks. Maria Montero Mirabet reports a relationship with Evonik Industries AG that includes: employment and equity or stocks. Jian-Feng Zhang reports a relationship with Evonik Industries AG that includes: employment and equity or stocks. Christoph Krauss has patent pending to Evonik Industries AG. Maria Montero Mirabet has patent pending to Evonik Industries AG. If there are other authors, they declare that they have no known competing financial interests or personal relationships that could have appeared to influence the work reported in this paper.

## Data Availability

Data will be made available on request.

## Appendix A Supplementary data

Supplementary material: SEM pictures and size measurement of DMTMM MR 1:0.08, DMTMM MR 1:0.04 and control sample in dry and wet state. Image 1
