# Supplementary material for: Electrospinning of animal-free derived collagen-like protein: Development and characterization of VECOLLAN®- nanofibers for biomedical applications
Source: Int J Pharm X. 2025 Sep 19;10:100398. doi: 10.1016/j.ijpx.2025.100398 (PMC12510221; doi:10.1016/j.ijpx.2025.100398)
Supplement: Supplementary file 1 — Supplementary material: SEM pictures and size measurement of DMTMM MR 1:0.08, DMTMM MR 1:0.04 and control sample in dry and wet state. [file mmc1.docx]

**Supplementary material**


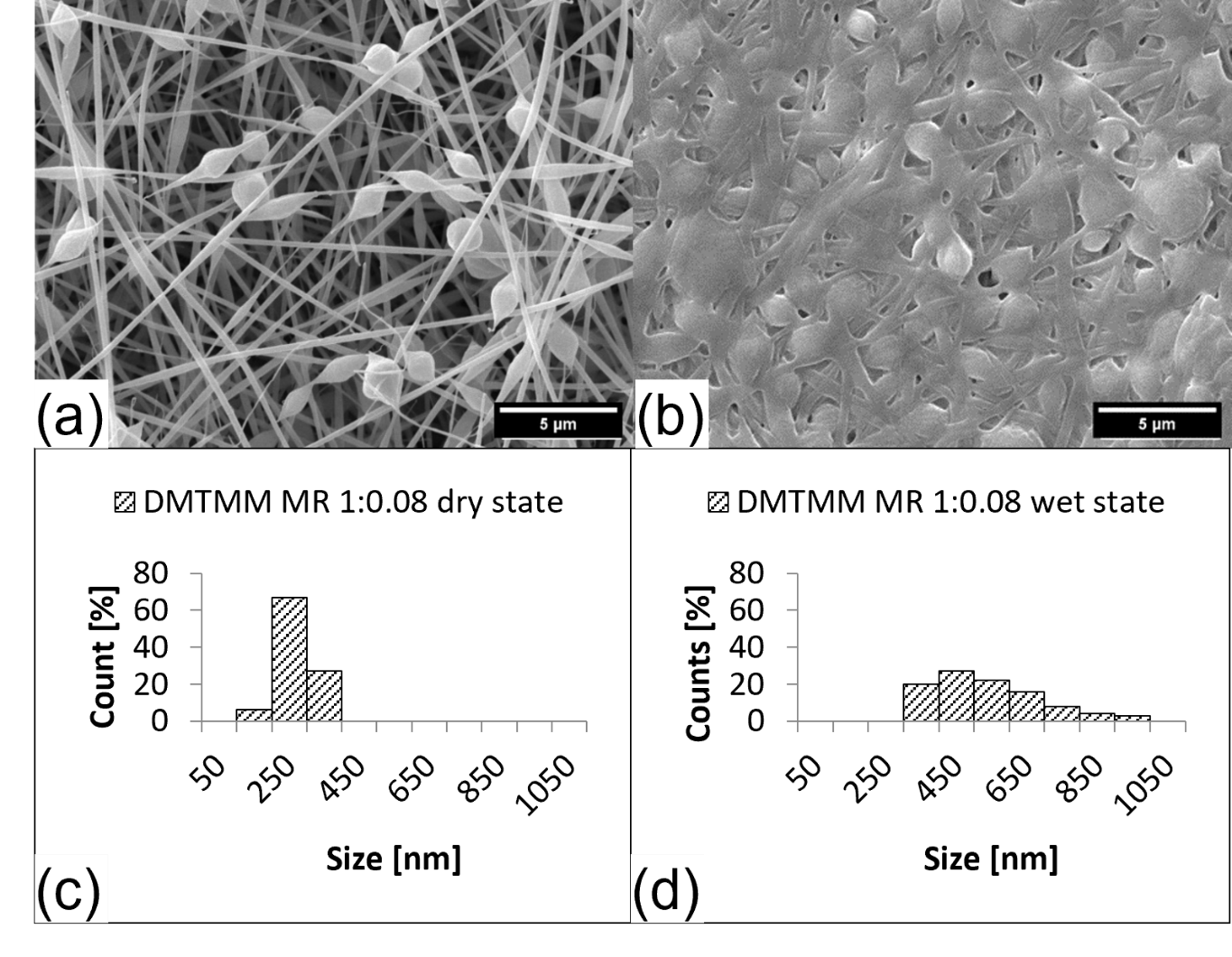


Figure S1: SEM pictures and fiber size distribution of crosslinked VECOLLAN®/PEO fibers combined with DMTMM (Molar ratio 1:0.08). (a) Dry state, (b) Wet state, (c) Average diameter: 275.3 nm, Standard deviation: 51.8 nm, Median: 274.1 nm, (d) Average diameter: 537.4 nm, Standard deviation: 148.2 nm, Median: 517.0 nm.


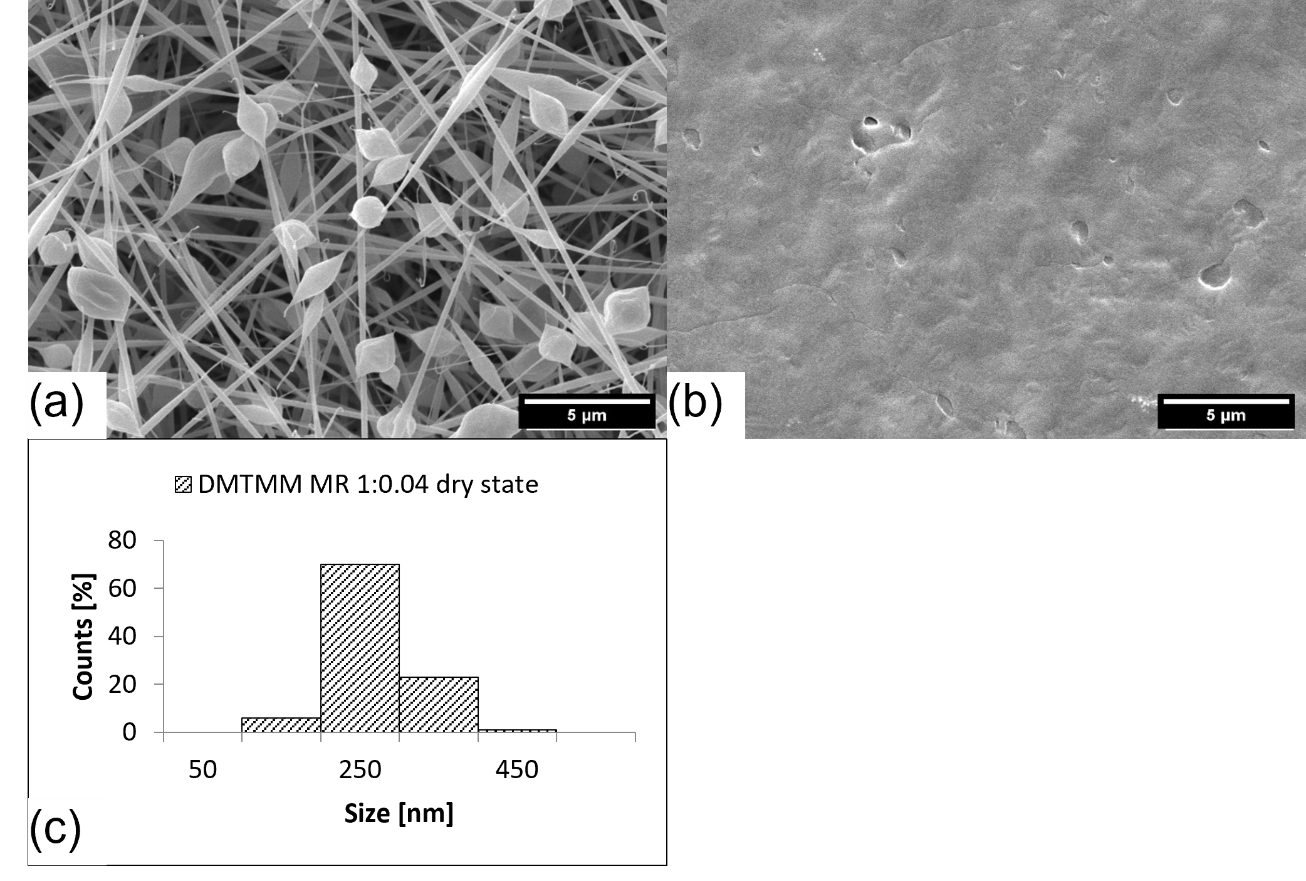


Figure S2: SEM pictures and fiber size distribution of crosslinked VECOLLAN®/PEO fibers combined with DMTMM (Molar ratio 1:0.04). (a) Dry state, (b) Wet state, (c) Average diameter: 273.6 nm, Standard deviation: 44.7 nm, Median: 269.7 nm. No measurement was possible for the wet state.


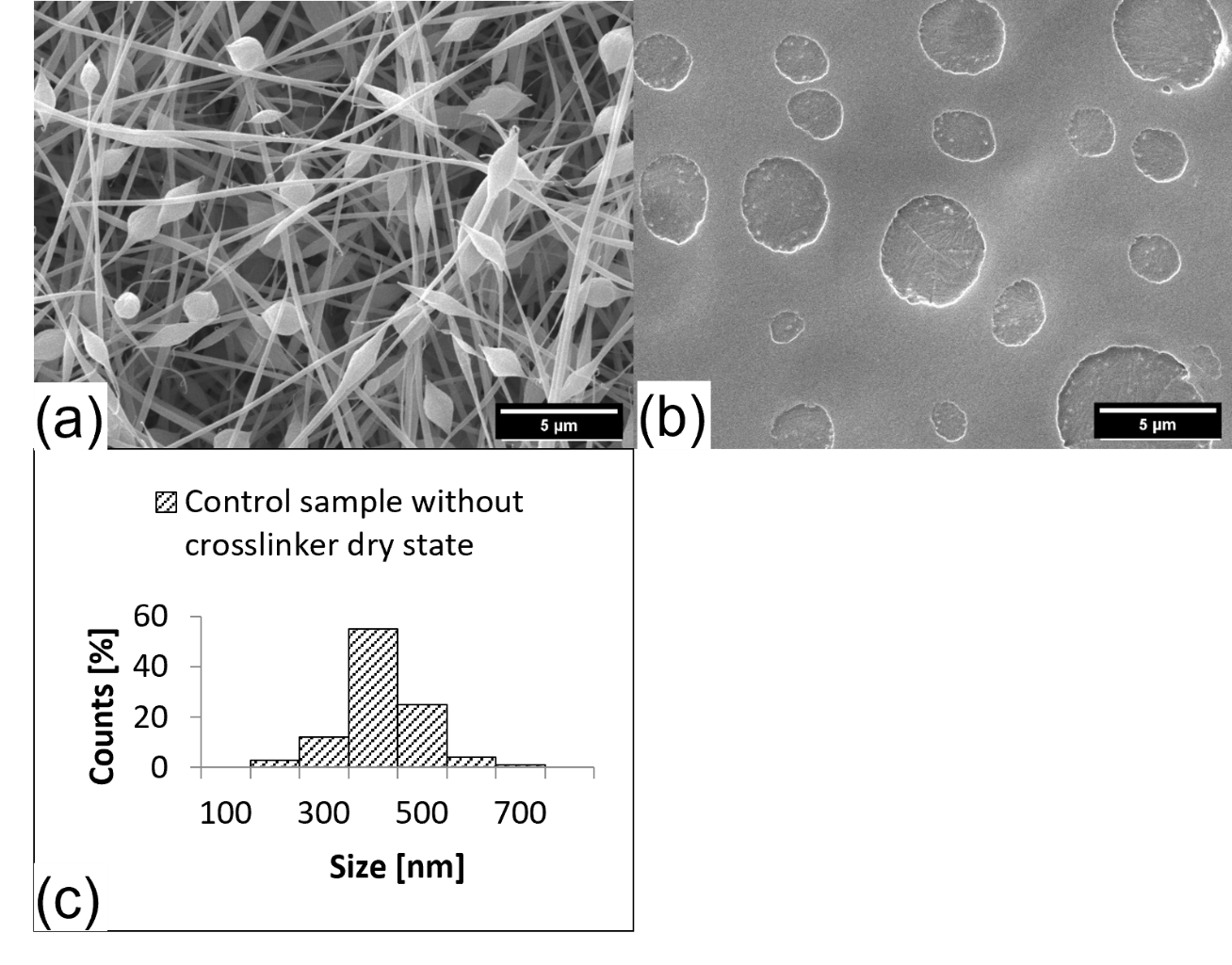


Figure S3: SEM pictures and fiber size distribution of crosslinked VECOLLAN®/PEO fibers without crosslinker. (a) Dry state, (b) Wet state, (c) Average diameter: 420.0 nm, Standard deviation: 98.2 nm, Median: 401.3 nm. No measurement was possible for the wet state.
